# Are There Circadian Clocks in Non-Photosynthetic Bacteria?

**DOI:** 10.3390/biology8020041

**Published:** 2019-05-22

**Authors:** Francesca Sartor, Zheng Eelderink-Chen, Ben Aronson, Jasper Bosman, Lauren E. Hibbert, Antony N. Dodd, Ákos T. Kovács, Martha Merrow

**Affiliations:** 1 Institute of Medical Psychology, Medical Faculty, LMU Munich, 80336 Munich, Germany; francesca.sartor@med.uni-muenchen.de (F.S.); chen.zheng@med.uni-muenchen.de (Z.E.-C.); 2 Department of Biology, University of Redlands, Redlands, CA 92373, USA; ben_aronson@redlands.edu; 3 Bioinformatics, Hanzehogeschool Groningen, 9747 AS Groningen, The Netherlands; j.bosman@pl.hanze.nl; 4 School of Biological Sciences, University of Bristol, Bristol BS8 1TQ, UK; lh14225@bristol.ac.uk; 5 Bacterial Interactions and Evolution Group, Department of Biotechnology and Biomedicine, Technical University of Denmark, 2800 Kgs. Lyngby, Denmark

**Keywords:** circadian, rhythm, clock, Eubacteria, entrainment, microbiome, temporal mutualism, holobiont

## Abstract

Circadian clocks in plants, animals, fungi, and in photosynthetic bacteria have been well-described. Observations of circadian rhythms in non-photosynthetic Eubacteria have been sporadic, and the molecular basis for these potential rhythms remains unclear. Here, we present the published experimental and bioinformatical evidence for circadian rhythms in these non-photosynthetic Eubacteria. From this, we suggest that the timekeeping functions of these organisms will be best observed and studied in their appropriate complex environments. Given the rich temporal changes that exist in these environments, it is proposed that microorganisms both adapt to and contribute to these daily dynamics through the process of temporal mutualism. Understanding the timekeeping and temporal interactions within these systems will enable a deeper understanding of circadian clocks and temporal programs and provide valuable insights for medicine and agriculture.

## 1. Introduction

Circadian clocks are molecular programs that impart temporal organization. These pervasive biological systems are responsible for distinct and characteristic patterns of daily activities that are observed throughout nature. Across the Kingdoms of life, circadian clocks have a broad influence upon cells, as evidenced by molecular rhythms in RNA abundance, protein abundance, protein modifications, metabolism; upon the organism, as evidenced by a set of stereotyped behaviors related to self-sustained rhythms and their synchronization; upon species, as evidenced by circadian rhythms in populations. Although circadian clocks are seemingly ubiquitous, they have rarely been described in non-photosynthetic prokaryotes. Here, we review those descriptions and speculate on the potential existence and nature of circadian systems in this group of organisms.

**Figure 1:**
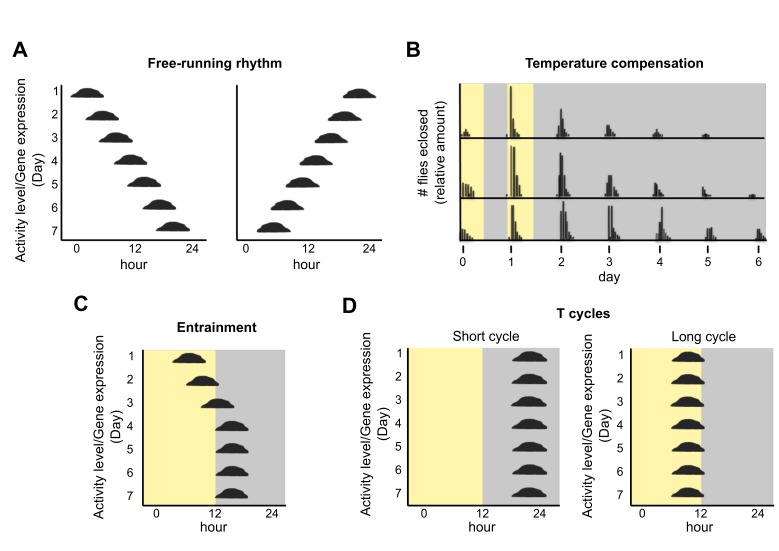
Characteristics of circadian clocks (see also [6]). (**A**) A free-running rhythm in constant conditions can have a period that is either slightly longer (left panel) or slightly shorter (right panel) than 24 h. Graphing the activity/amount (y-axis) over the course of the day (x-axis), such that changes from day to day are visually obvious (seen from top to bottom), facilitates an implicit understanding of period and phase of the circadian rhythm. (**B**) The eclosion of *Drosophila* occurs once per *ca*. 24 h in constant conditions following 2 days of entrainment in a 24 h light dark cycle (depicted as yellow and grey). The top of the panel shows eclosion at 26 °C, the middle shows 21 °C, and the bottom shows 16 °C. The timing of eclosion (and hence its period or frequency) remains almost the same over this broad range of physiological temperatures, and hence it is temperature compensated. Data are redrawn from Pittendrigh [7]. (**C**) The circadian rhythm entrains (synchronizes) to exactly 24 h in a zeitgeber cycle, shown here as a light-dark cycle. The rhythm gradually (days 1–4) finds a stable time of day (phase) relative to the zeitgeber (days 4–7). (**D**) T cycles are an experimental paradigm used to elucidate a biological clock. It places a rhythmic organism into entraining cycles of different lengths. The stable phase depends on the relationship between the period of the biological rhythm and the zeitgeber cycle. Most circadian rhythms entrain to a later phase in a shorter cycle and earlier phase in a longer one. Changes in phase angles in different T cycles are a hallmark of circadian clocks and occur regardless of the period length of the rhythm. These changes in phase angle distinguish circadian time—keeping from masking effects of the entrainment itself.

What is a circadian clock? The single observation that is most often used as proof of a circadian clock is a self-sustained, circa 24 h oscillation that continues in constant conditions (e.g., constant light or dark, constant temperature; Figure 1A). This is a remarkable feat that occurs in mammals, including humans, as well as in insects, plants, fungi, and several photosynthetic bacteria. These so-called free-running rhythms are, however, conditional. A mouse that has a free-running rhythm in constant darkness is arrhythmic in constant bright light. A gradual increase in light levels from darkness to moderate illumination causes a gradually-lengthening period of the mouse free-running rhythm with a concurrent decrease in the consolidation of activity/inactivity [1]. Similarly, a variety of rhythmic physiologies and underlying molecular processes cease to be rhythmic in *Arabidopsis* plants under constant darkness. Therefore, even model circadian clock systems with a robust and dependable circadian rhythm in a certain constant condition can be non-rhythmic in environments where a single variable is changed. In the absence of a good understanding of how an organism responds to its natural environment, it may be difficult to identify the correct set of conditions that would support robust, free-running rhythms.

Other rhythmic properties (Figure 1B,C) that are considered reliable evidence of a circadian clock include temperature compensation of the period of the free-running rhythm and synchronization of the rhythm to a 24 h environmental cycle or to appropriate stimuli (called zeitgebers, German for “time givers”). Temperature compensation refers to the stability of a free-running rhythm over a range of temperatures [2,3]. For circadian biology, the actual or imputed change in period length over 10 °C is calculated (the Q_10_). For circadian systems, this number is close to one (higher than one in most poikilotherms and lower than one in cells or tissues from homeotherms) [3,4,5]. For context, chemical reactions can have a Q_10_ of *ca*. 5, and cellular processes that are not temperature compensated (some enzymatic reactions) have a Q_10_ of 2–3.

Entrainment of the circadian rhythm to its cyclic environment (Figure 1C) leads to the establishment of a specific phase of the biological rhythm relative to the zeitgeber (the entraining agent for a biological clock) [6]. The endogenous oscillation (the circadian rhythm) actively probes the environment until it finds the optimal time point in the zeitgeber cycle where it no longer advances or delays but is stable, remaining at the same phase, until either the biological or environmental oscillator changes. Indeed, it is entrainment that creates the concept of “clock” for this temporal program because the oscillator combined with the zeitgeber cycle will show a characteristic, unique, entrained phase (chronotype) [7]. This property is thought to reflect the biological function of the circadian clock, namely to adaptively match function to a certain time of day.

A circadian rhythm alone, in a single condition, is not generally taken as solid proof of the existence of a circadian clock. Likewise, synchronization or entrainment of said rhythm to a single zeitgeber cycle in the absence of a free-running rhythm is generally not taken as evidence of a daily clock. It could simply be a driven response to the zeitgeber [8]. When do we start to believe that an endogenous clock is present in an organism? A circadian rhythm that is temperature compensated is compelling evidence because it shows that the temporal program in question has a built-in mechanism to protect against temperature effects that invariably occur and would otherwise disturb its timing function. Systematic entrainment properties, such as earlier or later phase angles in longer or shorter zeitgeber cycles (Figure 1D; T cycles), are likewise considered as evidence of a clock because they demonstrate an endogenous biological rhythm that is capable of direction—in this case, capable of directing the system to a certain phase angle relative to the entraining zeitgeber cycle as a result of entrainment by an oscillator with a unique set of characteristics [9,10]. A driven (non-entrained) rhythm would not show a systematic set of phase angles relative to an entraining cycle but rather uniform ones. These concepts are exemplified by the circadian system in *Neurospora crassa*. Light drives rapid expression of the RNA of clock gene *frequency* at any time of day whereas the FREQUENCY protein is produced at a phase that correlates with mid-dark or mid-light phase rather than the time when the lights turn on or off [11]. The outward behavior (spore formation) also correlates with the midpoint of the dark or light phase rather than the transitions between them [12]. These characteristics of rhythms have rarely been demonstrated outside of the eukaryotic Kingdom.

Among the prokaryotes, certain species of cyanobacteria provide clear evidence for a robust circadian clock, with *Synechococcus elongatus* representing the primary model system [13]. *S. elongatus* has one of the best characterized molecular clocks of all experimental models. Invoking the power of a prokaryotic model system, three essential clock proteins have been identified (KaiA, KaiB, and KaiC). These three proteins direct clock-regulated gene expression of almost the entire genome [14] (a much greater proportion of circadian transcription than with eukaryotic model systems). KaiA, KaiB, and KaiC are capable of circadian clock function at a purely post-transcriptional level through cycles of phosphorylation and de-phosphorylation as demonstrated by remarkable clock-in-a-tube experiments. The three purified Kai proteins combined with ATP lead to temperature compensated circadian rhythms that are capable of synchronization to 24 h temperature cycles [15]. When these proteins were transformed into *Escherichia coli*, they underwent a circadian oscillation in the phosphorylation of KaiC [16]. The KaiC protein, in the absence of KaiA and KaiB, is not rhythmically phosphorylated in *E. coli*, and thus the rhythmicity is derived from several cyanobacterial clock genes. The *S. elongatus* circadian clock has a temperature compensated free-running rhythm [17] and entrains with phase angles in T cycles [18]. Finally, *S. elongatus* is one of the very few examples that has been used to demonstrate that a circadian clock contributes to adaptive selection [18,19]. Experiments with otherwise fit clock mutant strains (e.g., with a short or long free-running period) with a wild type strain were cultured in constant conditions as well as in T cycles. When the length of the T cycle matched the period of one strain better than the other, that strain had superior fitness, whether it was a mutant or not. Furthermore, mutagenesis of “every” gene within the *S. elongatus* genome identified a set of genes that contribute to fitness under light/dark cycles [20]. This includes genes associated with managing carbon fluxes over the day-night cycle, those involved in responses to light-induced stresses during the transition from dark to light, and also the gene encoding the oscillator component KaiA [20]. Therefore, the clock rapidly shapes microbial population structure under light/dark cycles, and cyanobacteria have specific mechanisms to adapt to the daily fluctuating environment.

Rhythmic processes have also been reported in purple bacteria, which are capable of photosynthesis. For example, *Rhodospirillum rubrum* has rhythms with a period of 12–15 h in enzymatic activity of the uptake hydrogenase (Hup), which participates in redox metabolism during photosynthesis and nitrogen fixation [21]. Similarly, under aerobic conditions, there are oscillations of transcription of the *aidB* gene in *Rhodobacter sphaeroides* with a period of 20.5 h that, intriguingly, gets shorten to 10–12 h under anaerobic conditions [22]. *aidB* encodes an acyl-CoA dehydrogenase. The genome of *R. sphaeroides* harbors orthologs of *kaiB* and *kaiC*. It lacks a KaiA homolog, the protein which promotes KaiC phosphorylation [22]. In *R. sphaeroides*, the *kaiBC* operon includes a Per-ARNT-Sim (PAS) domain protein, which is intriguing because PAS-domain proteins are often associated with circadian clocks in a variety of species. Although the rhythms in purple bacteria do not appear to meet the criteria for classification as a circadian clock, they demonstrate that other groups of bacteria can also possess endogenous temporal programs. We suggest that other microbes, even those that are non-photosynthetic, are subject to evolutionary pressure from zeitgeber cycles, leading to the development of endogenous temporal programs.

It has been argued previously that a circadian rhythm might not be adaptive for single-celled organisms that have reproductive times shorter than the diel cycle, meaning that there might not be the selection for circadian programs in a variety of microorganisms [23]. This has been referred to as the “circadian-infradian rule” [23]. However, experiments with *S. elongatus* refute this notion. Cyanobacteria with generation times of between 5 and 10 h have been found to retain circadian rhythms in gene expression [24]. In addition, a memory effect has been identified in non-photosynthetic bacteria, which spans over the cell cycle interval (i.e., the physiological state of a bacterium is sustained for several cell divisions) [25]. Therefore, we speculate that circadian clocks may exist within other bacterial species, including rapidly dividing non-photosynthetic bacteria, that are exposed to a rhythmic environment that imposes a selection pressure.

Here, we review the published evidence that suggests circadian rhythms can exist in non-photosynthetic Eubacteria whose doubling times can be shorter than the diel cycle, review the bioinformatical hints that clocks might exist in such organisms, and provide reasoning for the historical and current challenges in identifying and describing a circadian timekeeper in these organisms.

## 2. Endogenous Circadian Rhythms in Non-Photosynthetic Prokaryotes

**Figure 2:**
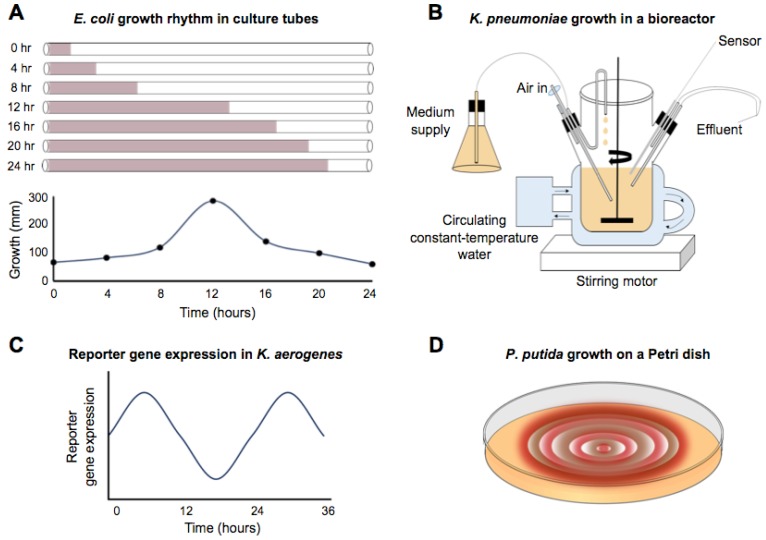
Schematic representations of the various rhythms observed for non-photosynthetic Eubacteria. (**A**) Growth rhythms observed in *E. coli* by growth in a long culture tube filled with liquid media and inoculated at one end. A dye was included to measure the growth front as the culture moves down the tube free-running rhythm. The graph summarizes the growth with 4 h intervals over 24 h, corresponding to hours 40 to 64 of the original experiment by Rogers and Greenbank [26]. Subsequent analysis of the data of the *E. coli* growth rhythm by Halberg and Conner suggests a 20.6 h period rather than 24 h [27]. (**B**) Sturtevant [30] measured the growth rhythm in *Klebsiella pneuomoniae* in a bioreactor. Samples were taken out every 15 or 30 min, and the optical density was measured. (**C**) Reporter gene rhythms detected in Klebsiella *aerogenes* [31]. A gene fusion between a motility gene (*motA*) and the reporter GFP allowed detection of circa 24 h rhythms. Similar types of long rhythms of reporter genes have been observed in *B. subtilis* [32,33,34,35]. (**D**) Morphological rhythms were observed by Soriano et al. [36] when studying *Pseudomonas putida* by incorporating dyes (Congo Red or Coomassie Brilliant Blue) that detect changes in the redox state or cell surface of *P. putida*. Growth “rings” were then observed that had circa 24 h rhythms.

The circadian-infradian rule is violated by the well-described circadian rhythms in *Synechococcus*, but there are also reports of circa 24 h period rhythms in non-photosynthetic bacteria that extend as far back as 1930 [26]. A 15 m long spiral growth tube filled with liquid media and a color indicator was inoculated at one end with *Escherichia coli*. The culture was incubated at 30 °C, and the growth front was measured approximately every hour over a period of 8 days. An “intermittent growth” (Figure 2A) was noted. Rogers and Greenbank [26] did not calculate or comment on the periodicity of growth, but their data showed long, circadian-like rhythms. Thirty years later, Halberg and Conner calculated the periodicity of the growth rate at 20.6 h [27]. Comparable studies were performed by Sturtevant [28], who monitored the growth of *E. coli* in a long tube of liquid culture, again using a color indicator to mark the growth front. During a 9.5-day experiment in darkness, with no apparent entrainment prior to or during the incubation, there was a 23.2 h rhythm in growth at 37 °C. A coincident 16 h rhythm was also reported, which might be a harmonic. When this experiment was repeated at 30 °C, rhythmic growth still occurred, although the period length no longer appeared to be circadian. *E. coli* grown at 30 °C had a 4.8 h ultradian rhythm. Therefore, these rhythms failed the criterion of temperature compensation of a free-running circadian rhythm. The idiosyncratic nature of these rhythms might also be supported by Bibb [29] who found no evidence of periodicity in the growth of *E. coli* under similar conditions. In this case, however, the time resolution of measurements is unclear, and the description suggests no active temperature control, which was reported as room temperature (24 °C–32 °C).

Reports of rhythmic growth of non-photosynthetic prokaryotic bacteria are not limited to *E. coli*. For example, rhythms have been identified in the growth of *Klebsiella pneumoniae* [30]. Rather than a tube configuration, this protocol used a fermenter that replaced the media at a rate of 0.25 h^−1^ under constant temperature and a 12:12 (fluorescent) light-dark cycle (Figure 2B). Following inoculation with *K. pneumoniae*, the optical density of the culture was measured at 15 or 30 min intervals. The pattern of optical density readings was fitted using a cosinor formula yielding a period of 24.1 h. No free-running rhythm was reported under constant conditions. Even under entrained conditions, the rhythm appeared to dissipate after about 4 days of the 13-day experiment.

More extended and robust observations of *ca*. 24 h rhythms have been reported in an enteric bacterium. Paulose et al. identified rhythms in the swarming response of *Klebsiella aerogenes* (called *Enterobacter aerogenes* in the publication) in the presence of melatonin [31]. *Klebsiella aerogenes* growth seemed uniquely sensitive to melatonin, in that several other species demonstrably lacked sensitivity to the hormone and *K. aerogenes* lacked sensitivity to melatonin-related molecules, such as tryptophan. A fusion of luciferase to a motility gene demonstrated that a pure culture of *K. aerogenes* had a ca. 24 h rhythm in constant conditions (Figure 2C) and that the rhythm was synchronized by the presence of melatonin. At 34 °C, a free-running rhythm persisted for four cycles. Furthermore, the authors showed that the rhythm was temperature compensated. In the presence of melatonin, the periodicity of the rhythm was about 25 h at temperatures ranging from 27 °C to 40 °C, with a reported Q_10_ of 0.96. This report is the clearest case of a classically defined circadian rhythm in a non-photosynthetic prokaryote: temperature compensated, free-running rhythms. It is noted by the authors that only about 35% of the cultures showed rhythmicity from the *MotA::luxcdabe * reporter, suggesting the conditional nature of the rhythm.

There is some evidence for daily rhythms under in vitro conditions in bacteria that inhabit the soil and can interact with plant roots. For example, Soriano et al. [36] showed that *Pseudomonas putida* had rhythmic growth on agar plates containing either Congo Red or Coomassie Blue, both of which interact with the extracellular matrix (Figure 2D). Under entrained conditions of 16L:8D or 12L:12D, the growth rhythms had a periodicity of about 24 h for both dyes (Congo Red, 24.1 h, Coomassie Blue, 24.9 h). When the cultures were shifted to constant conditions, the rhythm persisted for one cycle with a period of 19.2 h or 24 h (Congo red or Coomassie blue, respectively). No experiments were carried out to test temperature compensation of the rhythms.

The dyes that were utilized to show rhythms in *Pseudomonas putida* are differentially incorporated according to developmental state. Indeed, as they grow and develop, these organisms profoundly change their characteristics. Bacteria utilize complex regulatory pathways in these processes. Potentially, a circadian system might temporally organize the switching between different bacterial cell types, selecting the times of day that are most favorable and sensing environmental information to make costly switches in cellular biochemistry most efficient.

## 3. Possible Timekeeping Components in Non-Photosynthetic Eubacteria

Given these reports of apparent circadian rhythms in non-photosynthetic Eubacteria, is there evidence that there are timekeeping components in these organisms? It is tempting to make assumptions about function based on gene homology. This is certainly justified in the circadian clocks field where the Period gene is broadly involved in circadian timing in metazoans. Therefore, we would be remiss to not explore the occurrence of orthologous genes or clock-associated protein domains in non-photosynthetic bacteria as an indication of potential circadian systems. Dvornyk et al. [37] and Loza-Correa et al. [38] searched for homologs of *kai* genes in other bacteria. They found that *kaiA* is only present in cyanobacteria. Homologs of *kaiC* are found in three taxa of Bacteria (Proteobacteria, Thermotogae, and Chloroflexi). Interestingly, some Proteobacteria form close associations with organisms with identified circadian rhythms. One such example is the nitrogen-fixing rhizobacterium *Sinorhizobium medicae*, which possesses a *kaiC* homolog. *S. medicae* form root nodules on plants in the *Medicago* genus in the legume family [39,40]. *P. putida* also forms associations with plants, taking up secreted nutrients while providing protection against pathogens [41]. The *P. putida* genome has a *kaiC* homolog.

In addition to Kai homologs as possible clock components in non-photosynthetic Eubacteria, peroxiredoxin proteins may also be strong candidates. These proteins have circadian rhythms of their redox state in a wide variety of organisms, including in photosynthetic prokaryotic organisms like cyanobacteria and in the Archaea species *Halobacterium salinarum* [42]. These redox rhythms persisted in several species even when the previously defined major oscillator had been disabled. This led to the suggestion that the cycling of peroxiredoxin oxidative state is a separate but interconnected circadian clock in these organisms. Furthermore, the conserved nature of the peroxiredoxin proteins begs the question of whether the oscillation in their redox state might be a conserved oscillator that exists even in non-photosynthetic Eubacteria. According to Hall et al. [43], only *Borrelia* species lack evidence of the peroxiredoxin proteins. Thus, the examination of an entrained and a free-running rhythm of peroxiredoxin redox state in non-photosynthetic Eubacteria might provide further evidence of a circadian timekeeping mechanism in these organisms. Formally, and speculatively, these proteins might also be candidates of the oscillator itself.

In addition to a molecular mechanism that supports daily rhythms, circadian clocks must also have appropriate sensory pathways for processing zeitgeber inputs, such as light and temperature, for their entrainment. In free-living non-photosynthetic bacteria, one candidate zeitgeber molecule is bacteriophytochrome, a family that senses red and far-red light. These exist in a range of bacteria, including non-photosynthetic species, such as *Pseudomonas aeruginosa*, *Agrobacterium tumefaciens*, and *Deinococcus radiodurans* [44,45]. Many of the *Rhizobiaceae*, which form plant symbionts, have bacteriophytochromes [46] despite their occupation in the soil environment. Perhaps they sense light near the soil surface or, alternatively, might capitalize upon the light pipe function of some roots [47,48]. In either case, the potential exists for these bacteria to use light as a zeitgeber.

There is also evidence for functional blue light photoreceptors in non-phototrophic bacteria. The description of the light, oxygen, voltage (LOV) domain in *Bacillus subtilis* was the first documentation of this sensory motif outside of plants [49]. Since then, a repertoire of other blue light sensing photoreceptors has been identified in bacterial families, including the photoactive yellow protein (PYP) and BLUF (blue light sensing using FAD) domain proteins [50,51,52]. These domains are structurally related to each other and have similarities to the PAS domain. Many of these blue light sensing bacteria form associations with plant roots in the soil. Examples include the beneficial rhizobacterium *Burkholderia phytofirmans* that possesses genes with a PYP domain, and *B. subtilis* with a LOV domain within the blue light photoreceptor protein YtvA [53]. The pea-nodulating endosymbiont *Rhizobium leguminosarum* uses LOV domain light receptors to regulate exopolysaccharide production and the amount of root nodulation; however, it is also hypothesized that light sensing could allow rhizobia to sense the time of day and position within the environment to optimize root infection [54].

We note the confluence of three phenomena: non-photosynthetic Eubacteria (e.g., *E. coli*, *P. putida*, *K. pneumonia*, and *K. aerogenes*) with demonstrated rhythms do not yet meet classical circadian clock definitions, the prediction of potential clock components, such as KaiC and LOV domain proteins in these organisms, and the association of these bacteria with hosts that have a well-described circadian clock. Perhaps a key to better understanding timekeeping in non-photosynthetic Eubacteria is a model system that goes beyond a monoculture of the bacteria.

## 4. *Bacillus subtilis*—A Potential Model to Investigate Circadian Systems in Non-Photosynthetic Bacteria

The existence of circadian clocks in non-photosynthetic bacteria is currently speculative. However, the body of findings described above suggests that this might be worth revisiting. In considering promising model systems, we found several sporadic examples suggesting cyclic changes in gene expression, with a periodicity of the order of 24 h, in the Gram-positive bacteria *B. subtilis*. This bacterium lives in the soil, where it interacts with other organisms, including other bacteria [55,56,57], fungi [58], single cell predators (e.g., amoebae [59]), worms [60,61], and plants. In addition to biotic interactions, *B. subtilis* growth and differentiation is influenced greatly by changes in abiotic factors, including temperature. Under laboratory conditions, *B. subtilis* adaptation includes various modes of motility (single cell swimming, multicellular swarming, and sliding), natural competence for the ability to take up extracellular DNA that promotes genetic variability, protease production for degrading food sources, matrix production for biofilm formation that facilitates attachment and structure formation and, under adverse conditions, formation of highly resistant spores [62,63,64].

These differentiation processes are connected by complex regulatory pathways, including feedback loops, protein phosphorylation, and protein degradation. For instance, Süel et al. [32] used time-lapse fluorescence microscopy to follow the expression of genes determining the transient differentiation of *B. subtilis* into a competent state. This is the state where the bacterium can take up exogenous DNA from the environment. The entrance and exit from the competent state are controlled by an excitable core module containing positive and negative interactions determined by the cross-regulated expression of the *comK*, *comG*, and *comS* loci. ComK is the master regulator of competence state, and its proteolytic degradation is inhibited by the small ComS protein. When ComK protein approaches a threshold level in the cells, autoregulation boosts the production of ComK, which can activate later stage competence genes, including the *comG* operon. During competence, the activity of the promoters of *comK* and *comG* genes is highly correlated, while those of *comG* and *comS* are expressed anti-phase to one another. The promoter activity of these three elements shows an approximately 24 h oscillation under certain conditions [32]. While the cross-regulation between these components determines their relative change in abundance, one could speculate that a timekeeping system might underlie the periodicity of their expression.

Another example of ca. 24 h changes occurs during differentiation towards matrix production of *B. subtilis* under constant conditions that normally do not induce biofilm formation. Functionally, biofilm makes *B. subtilis* resilient in adverse conditions and also facilitates attachment to the root rhizosphere [65]. Norman et al. [33] monitored the expression of motility genes, by coupling the promoter of the *hag* gene encoding the flagellin subunit of the peritrichous flagellum to the red fluorescent mKATE2 reporter. They also monitored the expression of matrix genes in *B. subtilis* at a single cell level, by placing the blue fluorescent protein-coding gene *cfp* under the control of the *tapA* promoter and using a microfluidic device [33]. This setup allowed the observation of a single cell for several days and the activation of anti-phased expression of motility and matrix production pathways. The activation of matrix production and coupled chain formation occurred for only 7–8 generations of a cell in the absence of biofilm inducing signals. Intriguingly, the activation of matrix gene expression pulsed with an approximately 24 h period, suggesting that even under homogeneous conditions, the stochastic triggering of biofilm formation follows an internal cycle of this bacterium. Detailed analysis of this random initiation of matrix gene expression revealed that noisy antagonism within the complex regulatory pathway determines the chance of activation.

Yet another process that appears rhythmic in *B. subtilis* is spore formation, a well-documented clock-regulated process in *N. crassa* [3]. The formation of highly resistant spores in *B. subtilis* depends on cell physiology (i.e., starvation) and environment [62]. The initiation of the spore development depends on the global regulator Spo0A, the activity of which is influenced by a phosphorelay. During sporulation, the forespore encompasses the whole bacterial chromosome and is subsequently decorated by a spore coat that enables the packing of genetic material [66]. The timing of this process was dissected, showing coordination of spo0A expression with DNA replication [67,68]. On the one hand, the timed expression of feedback from the phosphorelay activating kinase (KinA) towards Spo0A phosphorylation ensures the cyclic but continuous increase of Spo0A with each cell cycle [30]. On the other hand, the genomic arrangement of the genes involved in the phosphorelay leads to a transient gene dosage imbalance during chromosome replication that contributes to the coordinated pulsatile expression of Spo0A phosphorylation [68].

Cyclic changes in the Spo0A pathway also occur in a cell cycle-independent manner. Single-cell tracking of *B. subtilis* during the early progression to spore formation identified bursts of promoter activity of several genes with a 20 h period. These genes encode the receptor aspartyl phosphatase A (RapA, a negative effector of the phosphorelay) and the phosphotransferase Spo0F. These are key components of the phosphorelay signal transduction pathway implicated in cell fate decisions between sporulation and growth of the bacterium [34,35]. While the sum of these observations does not define a circadian oscillator in *B. subtilis*, these observations do show the possibility of daily regulation of gene expression in pathways involved in sensing and integrating environmental changes in bacteria.

Our survey of the literature suggests that non-photosynthetic prokaryotes are capable of generating rhythmic gene expression with periods that are considerably longer than the typical cell division times in most laboratory conditions. The data are also remarkable for what seems to be missing; stability of period length, self-sustainment of the free-running rhythm, systematic entrainment to T cycles (apparently not investigated), and, with one exception, evidence of temperature compensation. Does the evidence suggest that non-photosynthetic bacteria have circadian clocks that adhere to historical definitions [69]? Or are these examples of less sophisticated timekeeping mechanisms, described by Johnson as proto-circadian oscillators [70]? If the former, perhaps laboratory conditions provide the equivalent to *B. subtilis* of constant dim light to a mouse: conditions that are incompatible with self-sustained, robust circadian rhythms. In the laboratory, the growth environment is most often reduced to simplicity. For example, a monoculture is typically grown in one of several standards, defined media in contrast to a noisy natural environment with complex signals derived from growth substrates and other microorganisms. Could the circadian properties be better observed under different conditions, for example, using media in which cell division occurs less frequently, or in conditions where nutrients are not exhausted quickly; for example, in fermenters? If the latter, why would bacteria—organisms that are constantly bombarded with changing environmental conditions stemming from the day/night cycles and capable of rapid genetic evolution—fail to have a demonstrable, robust circadian system? The analysis of microbiomes has been a revolution in microbiology. It is important to consider that microbes are virtually never found in isolation in nature, but rather in tremendously complex communities, including bacteria, fungi, and viruses. This may be an important observation because the description and rhythmic activities of microbiomes as a whole could be the key to circadian rhythms in non-photosynthetic bacteria.

## 5. Microbiomes and Daily Rhythms

Intestinal microbiomes are remarkable for their complexity, reaching about 1000 bacterial species in humans. Fifteen to twenty percent of the taxonomic units in the intestinal microbiome shift in an amount relative to one another each day [71,72,73]. This suggests the impact of the host on the gut bacterial content. By logical reasoning, this could be mediated either by behavior (ingestion of food at a certain time which would act via its food value for the bacteria), by the clock-regulated intestinal epithelium, or by endogenous core body temperature cycles. Restricted and controlled feeding in clock mutant mice supports the former mechanism, but the other factors are not ruled out.

There are many examples of rhythmic outputs from the gut microbiome impacting the host circadian clock. The amplitude of clock gene expression in the intestinal epithelium is altered in antibiotic-treated mice. *RevErbα*, *Per1*, and *Per2* transcript abundance increases while that of *Bmal1*, *Cry1*, and *RORα* decreases [74]. By changing the microbiome in a number of ways (diet, germ-free conditions), it has been demonstrated that the intestinal microbiome controls at least part of the rhythmic liver transcriptome [75,76,77]. In a remarkable experiment, disruption of the gut microbiome led to the retention of some daily rhythms in the gut and liver, abolished other rhythms in those tissues, and resulted in de novo expression of yet another cohort of rhythmic genes [78]. At least part of this set of regulated genes shows sexual dimorphism [79]. Furthermore, there are high amplitude changes in the number of bacteria associated with the intestinal wall according to the time of day [78]. This could be mediated by either the host (rhythmic chemokines) or by the microbial population (rhythmic responses to chemokines).

**Figure 3:**
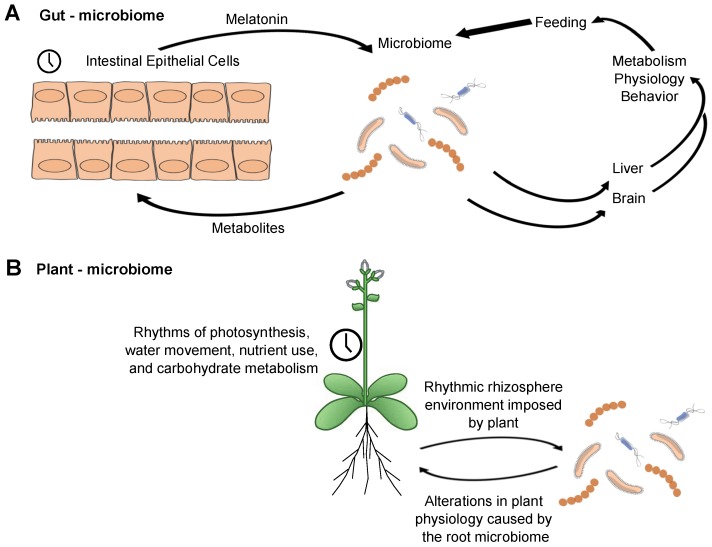
Interactions between circadian clocks and microbiome communities are established. (**A**) The clock in the host (here shown as the intestinal epithelial cells) shapes the gut microbiome community and the microbiome, in turn, impacts many clock-regulated outputs. (**B**) Similar results have started to appear relative to plant microbial communities. It is as yet unclear how much endogenous clock-like function the microbes may possess. We propose that the microbial community may utilize mutualism with a temporal structure in such a way that is consistent with the observations published thus far.

We, therefore, propose a model for the interaction of local clocks in the intestine that encompasses numerous observations (Figure 3A). The intestinal epithelial cells have a rhythmic expression of clock genes with an attendant set of clock-controlled outputs that will modify the local environment over the course of the 24 h day. They also are the source of intestinal melatonin [80]. Although we do not know whether the melatonin that they produce is rhythmic, there are rhythms in its direct precursor, serotonin [81]. Among other factors, melatonin will be utilized by at least some of the bacteria, possibly as a zeitgeber. The microbiome, in turn, will secrete metabolites reflecting daily rhythms in the feeding of the host, inputs from the epithelium—and possibly endogenous bacterial timing programs, though this is not an essential feature of the model. The metabolites can be used in a number of ways: they might feed back to the intestinal epithelium; they could be utilized among the bacterial population, signaling within the community; they clearly can impact peripheral liver clock-regulated genes. An important connection to make will be if the presence of a circadian rhythm in the gut (and its microbiome) impacts the clock in the brain. The gut-brain axis is clearly established, though how the clock might regulate this remains unknown. Clock-controlled outputs from the gut microbiome to the brain may form a feedback loop if they influence the timing of feeding behavior.

If gut microbial communities serve a function within a circadian system, then other well-characterized microbiomes/host arrangements might be expected to share some of the same principles. To that end, interactions between the root microbiome and circadian rhythms in plants have been reported. Clock regulation has a pervasive influence upon the physiology and development of plants, regulating processes ranging from the season of flowering to daily cycles of photosynthesis, stomatal opening, energy supply during the night, and fitness [82,83,84,85,86]. Cultivation of *Arabidopsis* short or long period clock mutants on soil led to alterations in the microbiome composition compared with the composition of the microbiome surrounding wild type plant roots [87]. Conversely, wild type plants had decreased leaf rosette diameter and delayed germination when cultivated on soil that was previously used to grow circadian clock mutants. One potential explanation is that mutants with an altered free-running period associate an altered microbiome, and this particular microbial community provides fewer benefits to the plant (here, assessed as from ‘conditioned’ soil) [87].

As with the mouse gut microbiome, the plant microbiome can alter the host circadian rhythms. Application of filtered soil samples that were either untreated or manipulated to disrupt the microbiome (sterilized or filter-sterilized) to tissue-cultured *Arabidopsis* seedlings led to differential effects upon their free-running period [87]. In another experimental protocol, the circadian period of *CCA1:LUC* in *Arabidopsis* is shortened by application of the bacterial flagellar peptide flg22 [88]. Although this example relates to plant defense mechanisms, it provides a proof of concept that plant surface microbes can alter plant circadian rhythms. Thus, as with the mouse intestinal microbiome, the plant/soil microbiome shows bidirectional interactions. Although some differences will surely be employed by the plant and animal microbial communities, similar features are apparent between the two systems. The intersection of circadian clock regulation, microbiome, and the host may follow a set of common principles.

## 6. The Microbiome as an Organism and Temporal Structuring of Mutualism

Recent developments in chronobiology research show circadian clocks as complex systems. Within a single cell, there exists a complex, multi-feedback loop network that comprises the system, stretching across all levels from transcription to metabolism. However, these cells function not so much as individuals but together as organs, which then must coordinate daily timing between each other. The clock is an evolved system that necessarily involves coordination between partners on many scales [6,89]. This concept may help in dissecting circadian clock properties in the microbiome.

How is the organization of the gut microbiome like a higher-level organism? Mice, for example, take synchronizing signals at the behavioral level from the environment (zeitgeber cycles). The gut microbiome must take temporal signals from its environment. These signals are coming from the host-temperature cycles, behaviorally-regulated feeding, the intestinal epithelium, and also from the surrounding microbial neighborhood. As microbes respond to timed signals, such as periodic food supplies or higher or lower temperatures or endogenous hormone levels, they will adjust their physiology (Figure 3). If these signals are highly predictable, the organism will evolve a system to prepare for the regular input, the first step to an endogenous temporal structure. The signals will function as zeitgebers.

Indeed, the microbiome shows organized localization of communities of a given microbe accordingly over time. At some times of day, certain species are intimate with the gut wall [78]. At other times, they are more internal to the lumen. This suggests that there is coordination at the species level over time. Therefore, the gut-microbiome interactions occur across the three dimensions of space as well as the fourth dimension, time.

What we have described is essentially a variation of the holobiont concept [90,91], namely that individual organisms deliver emergent properties when observed within their endogenous community. We suggest that daily time, with its pervasive, extensive structure of cues and signals, is an essential element for microbe-rich holobionts. This might be the case, for example, in microbial mats, where rhythmic gene expression from photosynthetic microorganisms is evident, but so is rhythmic gene expression from non-photosynthetic bacteria that are also part of the mat [92]. A simple mechanism whereby this may be implemented is cross-feeding. Cross-feeding is emerging as a major aspect of microbial biology that is thought to explain the inability to isolate certain species as individuals in vitro [93]. The organism benefits from coordinating resources with neighbors, as it can effectively shut off costly metabolic pathways. The ideal neighborhood would feature a collection of complementary producers. Several metabolites that are cross-fed by bacteria are circadian clock-regulated in their presence as well as in their regulatory proteins in eukaryotic clock systems (e.g., lactate in mice).

Bringing these ideas together, we propose the novel concept of **temporal mutualism,** defined as rhythmically structured metabolic exchanges between microbes for mutual benefit within a community. Temporal mutualism may, therefore, underlie rhythmic emergent behavior within the community and have acted to select for timing systems in non-photosynthetic bacteria.

## 7. Why Do We Care?

The potential for circadian rhythms in the Eubacteria is a big deal. Bacteria play a crucial role in medical, agricultural, and industrial processes. Knowledge of potential microbial circadian rhythms would allow us to capitalize on the rhythms to tune how we interact with bacteria in all of these areas.

The presence of a circadian clock in microbes could impact health problems stemming from or associated with microbes. One of the crises in medicine at this time is the prevalence of antibiotic-resistant bacteria (MRSA). This reflects an antibiotic overuse, creating an evolutionary pressure on the bacteria to evolve escape mechanisms, as well as a deficit of new antibiotics. Application of new antibiotics would simply delay the problem until new escape tricks are evolved. The combination of invoking bacterial circadian clocks such that they slow down metabolism or replication paired with time-of-day specific administration of antibiotic may be a more powerful form of treatment, potentially delaying development of resistance. The escape drive would have to focus on multiple targets, including the timing system. Concerning the biology of the infection itself, the humoral immune system, experimentally characterized by how an organism responds to lipopolysaccharide, a component of the bacterial cell wall, has a high amplitude daily rhythm [94]. Administration of 20 mg/kg to mice at ZT 6 (night time for a mouse) leads to death of 50% of the animals within 24 h. Almost all mice treated at ZT18 (in the middle of the active period) are alive after 3 days. Control of exposure times may lead to more or less successful infections.

There is an indication that viral infection and response to infection is a time of day specific. The entry of viral particles of flaviviruses (RNA viruses, including Dengue and Zika) into cells, as well as the replication of their genome, is regulated by clock genes [95]. Infection of mice with Herpesvirus (a DNA virus) at the beginning of the light phase leads to a higher viral load relative to infection at the end of the light phase [96]. This outcome is dependent on a functional BMAL1 protein. It is unlikely that the virus has an active clock, but it may borrow or plug into host temporal structures for optimal function.

The strongest case for the impact of a microbial circadian clock on health is emerging from the gut microbiome. Although we still do not know if the gut microbiota possesses endogenous circadian clocks, the interaction of host clock with microbe, the association of clock dysfunction with disease states, and the association of particular microbial populations with disease make it tempting to speculate on how microbial circadian clocks may be involved in maintaining health or transitioning to disease. If microbes are passive players, simply responding to host synchronization, then attending mainly to supporting the host clock should restore a healthy microbiome structure. If the microbiome has a clock of its own, then it will follow the rules of entrainment. The amplitude of rhythms, for instance, would be expected to decrease in phase shifting protocols [97], in contrast to a clock-less driven system.

In business and industry, bacteria are used for qualitative and quantitative production. Invoking the circadian clock—working with the rhythmic physiology of the producers—could increase production, as well as improve the quality of the product [98]. Perhaps the most common application is fermentation, which traditionally was performed in loosely controlled conditions (in the cellar or in a cabinet in a kitchen). Modern factory production strives for tightly controlled conditions, which may inadvertently be a source of either decreased productivity or alteration in flavors. We made sauerkraut (as well as *Saccharomyces cerevisiae*-fermented beer and rice wine) under strictly controlled temperature conditions, as well as under appropriate 24 h temperature cycles (unpublished results, Zheng Eelderink-Chen, Margien Raven, and Martha Merrow). The results were taste tested in single-blind experiments. In all cases, the subjects could distinguish the foods from the two protocols. Preference was mixed. The deliberate imposition of controlled zeitgeber (e.g., temperature) cycles on fermentation processes should adjust the metabolic pathways, thus leading to the accumulation of distinct metabolites, and hence leading to unique flavors [98].

Agriculture has been employing microbes to optimize crop production for some time. Root-colonizing bacteria (rhizobacteria) are beneficial for plant growth enhancement and agricultural disease suppression [99]. For example, *B. subtilis* forms associations with plant roots, contributing to the growth and health of crops. The nature of the interaction between plants and *Bacillus* spp. is complex and involves signaling between the plant and bacteria. The bacteria are initially attracted towards roots by the detection of root exudates, such as L-malic acid, in a process involving chemotaxis [100]. Subsequently, the bacteria form adhesive biofilms on the root surface [101]. Recognition of plant cell wall polysaccharides activates bacterial matrix genes, causing the bacterial synthesis of extracellular polymers that form a matrix between bacterial cells [102]. After colonization, the bacteria can utilize plant root exudates while benefiting the plant through growth enhancement and disease suppression. Examples of crop growth enhancement by *B. subtilis* include an increase in tomato root length and plant height by inoculation with *B. subtilis* PTS-394, increase in root elongation of chickpea seedlings of 70–74% after inoculation with *B. subtilis* strains CM1 and CM3, and increased leaf length, flowers per corm, and stigma biomass after inoculation of saffron (*Crocus sativus*) with *B. subtilis* FZB24 [103,104,105]. There are also many examples of enhanced crop protection from pathogens by inoculation with *B. subtilis*, such as antifungal activity against peach brown rot (*Monilinia* spp.) [106] protection against grey mold (*Botrytis cinerea*) of apple post-harvest, and against damping-off caused by *Pythium ultimum* in bean seedlings [107]. In addition to fungal pathogens, *B. subtilis* colonization of *Arabidopsis thaliana* confers protection against the major agricultural bacterial pathogen *Pseudomonas syringae* by secretion of the antimicrobial lipopeptide surfactin and the induction of systemic resistance [108].

Given the considerable agricultural and economic importance of rhizobacteria, understanding the mechanisms that underpin plant-bacteria interaction and growth enhancement is important for identifying new ways to sustainably enhance agricultural production. It is possible that circadian-regulated processes in plants or bacteria might contribute to these interactions. For example, plant circadian clocks respond to metabolites, phytohormones, and nutrient conditions [109,110,111], all of which may be altered by microbial colonization. On the other hand, plant circadian regulation of processes, such as photosynthesis and carbohydrate metabolism [80,81], might create a rhythmic host environment for the colonizing bacteria. Importantly, the presence of circadian rhythms within roots [112,113] provides considerable potential for circadian programs within the plant to impact attraction and colonization by beneficial bacteria. We also speculate that there might be circadian rhythms in root membrane transport processes, such as proton pumping, that impose a fluctuating environment upon root-colonizing bacteria, although this is as yet uncharacterized. These bidirectional interactions between plant and bacterial circadian rhythms may be analogous to host-bacteria interactions in the gut microbiome [72,73]. It is possible to envisage that circadian programs in root-colonizing bacteria might allow the bacterial colonies to coordinate their metabolism with rhythms derived from the host plant in root exudate secretion, in order to maximize the benefit of the bidirectional interaction.

## 8. A Perspective

The glimpses of circadian rhythms in non-photosynthetic Eubacteria are tantalizing. We suspect that the timekeeping abilities of such organisms are significant but that witnessing of those abilities might require systems in which these microbes are in more complex environments than where they are typically cultured. The interaction between the timekeepers in these complex environments will be important to understand for basic scientific reasons, as well as for medical and agricultural reasons.

## References

[1] Abraham D., Dallmann R., Steinlechner S., Albrecht U., Eichele G., Oster H. (2006). Restoration of Circadian Rhythmicity in Circadian Clock-Deficient Mice in Constant Light. J. Biol. Rhythms.

[2] Pittendrigh C.S., Caldarola P.C. (1973). General homeostasis of the frequency of circadian oscillations. Proc. Natl. Acad. Sci. USA.

[3] Gardner G.F., Feldman J.F. (1981). Temperature Compensation of Circadian Period Length in Clock Mutants of *Neurospora crassa*. Plant Physiol..

[4] Barrett R.K., Takahashi J.S. (1995). Temperature compensation and temperature entrainment of the chick pineal cell circadian clock. J. Neurosci..

[5] Zimmerman W.F., Pittendrigh C.S., Pavlidis T. (1968). Temperature compensation of the circadian oscillation in *Drosophila pseudoobscura* and its entrainment by temperature cycles. J. Insect Physiol..

[6] Merrow M., Spoelstra K., Roenneberg T. (2005). The circadian cycle: Daily rhythms from behaviour to genes. EMBO Rep..

[7] Pittendrigh C.S. (1954). On Temperature Independence in the Clock System Controlling Emergence Time in *Drosophila*. Proc. Natl. Acad. Sci. USA.

[8] Mrosovsky N. (1999). Masking: History, definitions, and measurement. Chronobiol Int..

[9] Eelderink-Chen Z., Mazzotta G., Sturre M., Bosman J., Roenneberg T., Merrow M. (2010). A circadian clock in *Saccharomyces cerevisiae*. Proc. Natl. Acad. Sci. USA.

[10] Aschoff J., Pohl H. (1978). Phase relations between a circadian rhythm and its zeitgeber within the range of entrainment. Naturwissenschaften.

[11] Tan Y., Dragovic Z., Roenneberg T., Merrow M. (2004). Entrainment dissociates transcription and translation of a circadian clock gene in *Neurospora*. Curr. Biol..

[12] Rémi J., Merrow M., Roenneberg T. (2010). A circadian surface of entrainment: Varying T, τ, and Photoperiod in *Neurospora crassa*. J. Biol. Rhythms.

[13] Kondo T., Tsinoremas N.F., Golden S.S., Johnson C.H., Kutsuna S., Ishiura M. (1994). Circadian clock mutants of cyanobacteria. Science.

[14] Liu Y., Tsinoremas N.F., Johnson C.H., Lebedeva N.V., Golden S.S., Ishiura M., Kondo T. (1995). Circadian orchestration of gene expression in cyanobacteria. Genes Dev..

[15] Nakajima M., Imai K., Ito H., Nishiwaki T., Murayama Y., Iwasaki H., Oyama T., Kondo T. (2005). Reconstitution of circadian oscillation of cyanobacterial KaiC phosphorylation in vitro. Science.

[16] Chen A.H., Lubkowicz D., Yeong V., Chang R.L., Silver P.A. (2015). Transplantability of a circadian clock to a noncircadian organism. Sci. Adv..

[17] Aoki S., Kondo T., Wada H., Ishiura M. (1997). Circadian rhythm of the cyanobacterium *Synechocystis* sp. strain PCC 6803 in the dark. J. Bacteriol..

[18] Ouyang Y., Andersson C.R., Kondo T., Golden S.S., Hirschie C. (2009). Resonating circadian clocks enh; ince fitness in cyanobacteria. Evolution.

[19] Woelfle M.A., Ouyang Y., Phanvijhitsiri K., Johnson C.H. (2004). The adaptive value of circadian clocks: An experimental assessment in cyanobacteria. Curr. Biol..

[20] Welkie D.G., Rubin B.E., Chang Y.-G., Diamond S., Rifkin S.A., LiWang A., Golden S.S. (2018). Genome-wide fitness assessment during diurnal growth reveals an expanded role of the cyanobacterial circadian clock protein KaiA. Proc. Natl. Acad. Sci. USA.

[21] Van Praag E., Agosti R.D., Bachofen R. (2000). Rhythmic activity of uptake hydrogenase in the prokaryote *Rhodospirillum rubrum*. J. Biol. Rhythms.

[22] Min H., Guo H., Xiong J. (2005). Rhythmic gene expression in a purple photosynthetic bacterium, Rhodobacter sphaeroides. FEBS Lett..

[23] Ehret C.F., Wille J.J., Halldal P. (1970). The Photobiology of Circadian Rhythms in Protozoa and other Eukaryotic Microorganisms. Photobiology of Microorganisms.

[24] Kondo T., Mori T., Lebedeva N.V., Aoki S., Ishiura M., Golden S.S. (1997). Circadian rhythms in rapidly dividing cyanobacteria. Science.

[25] Norman T.M., Lord N.D., Paulsson J., Losick R. (2015). Stochastic Switching of Cell Fate in Microbes. Annu. Rev. Microbiol..

[26] Rogers L.A., Greenbank G.R. (1930). The intermitent growth of bacterial cultures. J. Bacteriol..

[27] Halberg F., Conner R.L. (1961). Circadian organization and microbiology: Variance spectra and a periodogram on behavior of *Escherichia coli* growing in fluid culture. Proc. Minn. Acad. Sci. USA.

[28] Sturtevant R. (1973). Circadian patterns in linear growth of Escherichia coli. Anat Rec..

[29] Bibb L.B. (1925). Long Tube Method of Cultivating Microorganisms with Observations on Mobile Colonies in Liquid Medium. J. Bacteriol..

[30] Sturtevant R.P. (1973). Circadian variability in Klebsiella demonstrated by cosinor analysis. Int. J. Chronobiol..

[31] Paulose J.K., Wright J.M., Patel A.G., Cassone V.M. (2016). Human gut bacteria are sensitive to melatonin and express endogenous circadian rhythmicity. PLoS ONE.

[32] Süel G.M., Garcia-Ojalvo J., Liberman L.M., Elowitz M.B. (2006). An excitable gene regulatory circuit induces transient cellular differentiation. Nature.

[33] Norman T.M., Lord N.D., Paulsson J., Losick R. (2013). Memory and modularity in cell-fate decision making. Nature.

[34] Bischofs I.B., Hug J.A., Liu A.W., Wolf D.M., Arkin A.P. (2009). Complexity in bacterial cell-cell communication: Quorum signal integration and subpopulation signaling in the *Bacillus subtilis* phosphorelay. Proc. Natl. Acad. Sci. USA.

[35] Kuchina A., Espinar L., Çağatay T., Balbin A.O., Zhang F., Alvarado A., Garcia-Ojalvo J., Süel G.M., Çağatay T., Balbin A.O. (2011). Temporal competition between differentiation programs determines cell fate choice. Mol. Syst. Biol..

[36] Soriano M.I., Roibás B., García A.B., Espinosa-Urgel M. (2010). Evidence of circadian rhythms in non-photosynthetic bacteria?. J. Circadian Rhythms.

[37] Dvornyk V., Vinogradova O., Nevo E. (2003). Origin and evolution of circadian clock genes in prokaryotes. Proc. Natl. Acad. Sci. USA.

[38] Loza-Correa M., Gomez-Valero L., Buchrieser C. (2010). Circadian clock proteins in prokaryotes: Hidden rhythms?. Front. Microbiol..

[39] Cleyet-Marel J.C., Rome S., Salducci X., Wery J. (1996). Importance des composés phénoliques dans les interactions entre plantes et microorganismes: Exemple des relations Rhizobium/légumineuses. Acta Bot. Gallica.

[40] Rome S., Fernandez M.P., Brunel B., Normand P., Cleyet-Marel J.-C. (1996). *Sinorhizobium medicae* sp. nov., Isolated from Annual Medicago spp. Int. J. Syst. Bacteriol..

[41] Bernal P., Allsopp L.P., Filloux A., Llamas M.A. (2017). The Pseudomonas putida T6SS is a plant warden against phytopathogens. ISME J..

[42] Edgar R.S., Green E.W., Zhao Y., Van Ooijen G., Olmedo M., Qin X., Xu Y., Pan M., Valekunja U.K., Feeney K.A. (2012). Peroxiredoxins are conserved markers of circadian rhythms. Nature.

[43] Hall A., Sankaran B., Poole L.B., Karplus P.A. (2009). Structural Changes Common to Catalysis in the Tpx Peroxiredoxin Subfamily. J. Mol. Biol..

[44] Davis S.J., Vener A.V., Vierstra R.D. (1999). Bacteriophytochromes: Phytochrome-like photoreceptors from nonphotosynthetic eubacteria. Science.

[45] Bhoo S.H., Davis S.J., Walker J., Karniol B., Vierstra R.D. (2001). Bacteriophytochromes are photochromic histidine kinases using a biliverdin chromophore. Nature.

[46] Rottwinkel G., Oberpichler I., Lamparter T. (2010). Bathy phytochromes in rhizobial soil bacteria. J. Bacteriol..

[47] Lee H.J., Ha J.H., Kim S.G., Choi H.K., Kim Z.H., Han Y.J., Kim J.I., Oh Y., Fragoso V., Shin K. (2016). Stem-piped light activates phytochrome B to trigger light responses in *Arabidopsis thaliana* roots. Sci. Signal..

[48] Nimmo H.G. (2018). Entrainment of *Arabidopsis* roots to the light:dark cycle by light piping. Plant Cell Environ..

[49] Losi A., Polverini E., Quest B., Gärtner W. (2002). First evidence for phototropin-related blue-light receptors in prokaryotes. Biophys. J..

[50] Gomelsky M., Klug G. (2002). BLUF: A novel FAD-binding domain involved in sensory transduction in microorganisms. Trends Biochem. Sci..

[51] Tschowri N., Busse S., Hengge R. (2009). The BLUF-EAL protein YcgF acts as a direct anti-repressor in a blue-light response of *Escherichia coli*. Genes Dev..

[52] Memmi S., Kyndt J., Meyer T., Devreese B., Cusanovich M., Van Beeumen J. (2008). Photoactive yellow protein from the halophilic bacterium Salinibacter ruber. Biochemistry.

[53] Kumauchi M., Hara M.T., Stalcup P., Xie A., Hoff W.D. (2008). Identification of six new photoactive yellow proteins-diversity and structure-function relationships in a bacterial blue light photoreceptor. Photochem. Photobiol..

[54] Bonomi H.R., Posadas D.M., Paris G., del Carrica M.C., Frederickson M., Pietrasanta L.I., Bogomolni R.A., Zorreguieta A., Goldbaum F.A. (2012). Light regulates attachment, exopolysaccharide production, and nodulation in *Rhizobium leguminosarum* through a LOV-histidine kinase photoreceptor. Proc. Natl. Acad. Sci. USA.

[55] Müller S., Strack S.N., Hoefler B.C., Straight P.D., Kearns D.B., Kirby J.R. (2014). Bacillaene and Sporulation Protect *Bacillus subtilis* from Predation by *Myxococcus xanthus*. Appl. Environ. Microbiol..

[56] Shank E.A., Klepac-Ceraj V., Collado-Torres L., Powers G.E., Losick R., Kolter R. (2011). Interspecies interactions that result in *Bacillus subtilis* forming biofilms are mediated mainly by members of its own genus. Proc. Natl. Acad. Sci. USA.

[57] Gallegos-Monterrosa R., Mhatre E., Kovács Á.T. (2016). Specific *Bacillus subtilis* 168 variants form biofilms on nutrient-rich medium. Microbiology.

[58] Benoit I., van den Esker M.H., Patyshakuliyeva A., Mattern D.J., Blei F., Zhou M., Dijksterhuis J., Brakhage A.A., Kuipers O.P., de Vries R.P., Kovács Á.T. (2015). *Bacillus subtilis* attachment to Aspergillus niger hyphae results in mutually altered metabolism. Environ. Microbiol..

[59] Klobutcher L.A., Ragkousi K., Setlow P. (2005). The *Bacillus subtilis* spore coat provides “eat resistance” during phagocytic predation by the protozoan *Tetrahymena thermophila*. Proc. Natl. Acad. Sci. USA.

[60] Laaberki M.H., Dworkin J. (2008). Role of spore coat proteins in the resistance of *Bacillus subtilis* spores to *Caenorhabditis elegans* predation. J. Bacteriol..

[61] Donato V., Ayala F.R., Cogliati S., Bauman C., Costa J.G., Leñini C., Grau R. (2017). *Bacillus subtilis* biofilm extends *Caenorhabditis elegans* longevity through downregulation of the insulin-like signalling pathway. Nat. Commun..

[62] Lopez D., Vlamakis H., Kolter R. (2009). Generation of multiple cell types in *Bacillus subtilis*. FEMS Microbiol. Rev..

[63] Kearns D.B. (2010). A field guide to bacterial swarming motility. Nat. Rev. Microbiol..

[64] Hölscher T., Kovács Á.T. (2017). Sliding on the surface: Bacterial spreading without an active motor. Environ. Microbiol..

[65] Vlamakis H., Chai Y., Beauregard P., Losick R., Kolter R. (2013). Sticking together: Building a biofilm the *Bacillus subtilis* way. Nat. Rev. Microbiol..

[66] Higgins D., Dworkin J. (2012). Recent progress in *Bacillus subtilis* sporulation. FEMS Microbiol. Rev..

[67] Levine J.H., Fontes M.E., Dworkin J., Elowitz M.B. (2012). Pulsed feedback defers cellular differentiation. PLoS Biol..

[68] Narula J., Kuchina A., Lee D.Y.D., Fujita M., Süel G.M., Igoshin O.A. (2015). Chromosomal Arrangement of Phosphorelay Genes Couples Sporulation and DNA Replication. Cell.

[69] Pittendrigh C.S. (1960). Circadian rhythms and the circadian organization of living systems. Cold Spring Harb. Symp. Quant. Biol..

[70] Ma P., Mori T., Zhao C., Thiel T., Johnson C.H. (2016). Evolution of KaiC-Dependent Timekeepers: A Proto-circadian Timing Mechanism Confers Adaptive Fitness in the Purple Bacterium Rhodopseudomonas palustris. PLoS Genet..

[71] Zarrinpar A., Chaix A., Yooseph S., Panda S. (2014). Diet and feeding pattern affect the diurnal dynamics of the gut microbiome. Cell Metab..

[72] Liang X., Bushman F.D., FitzGerald G.A. (2015). Rhythmicity of the intestinal microbiota is regulated by gender and the host circadian clock. Proc. Natl. Acad. Sci. USA.

[73] Thaiss C.A., Zeevi D., Levy M., Zilberman-Schapira G., Suez J., Tengeler A.C., Abramson L., Katz M.N., Korem T., Zmora N. (2014). Transkingdom control of microbiota diurnal oscillations promotes metabolic homeostasis. Cell.

[74] Mukherji A., Kobiita A., Ye T., Chambon P. (2013). Homeostasis in intestinal epithelium is orchestrated by the circadian clock and microbiota cues transduced by TLRs. Cell.

[75] Montagner A., Korecka A., Polizzi A., Lippi Y., Blum Y., Canlet C., Tremblay-Franco M., Gautier-Stein A., Burcelin R., Yen Y.C. (2016). Hepatic circadian clock oscillators and nuclear receptors integrate microbiome-derived signals. Sci. Rep..

[76] Leone V., Gibbons S.M., Martinez K., Hutchison A.L., Huang E.Y., Cham C.M., Pierre J.F., Heneghan A.F., Nadimpalli A., Hubert N. (2015). Effects of diurnal variation of gut microbes and high-fat feeding on host circadian clock function and metabolism. Cell Host Microbe.

[77] Murakami M., Tognini P., Liu Y., Eckel-Mahan K.L., Baldi P., Sassone-Corsi P. (2016). Gut microbiota directs PPARγ-driven reprogramming of the liver circadian clock by nutritional challenge. EMBO Rep..

[78] Thaiss C.A., Nobs S.P., Elinav E. (2017). NFIL-trating the Host Circadian Rhythm—Microbes Fine-Tune the Epithelial Clock. Cell Metab..

[79] Weger B.D., Gobet C., Yeung J., Martin E., Jimenez S., Betrisey B., Foata F., Berger B., Balvay A., Foussier A. (2018). The Mouse Microbiome Is Required for Sex-Specific Diurnal Rhythms of Gene Expression and Metabolism. Cell Metab..

[80] Konturek S.J., Konturek P.C., Brzozowska I., Pawlik M., Sliwowski Z., Cześnikiewicz-Guzik M., Kwiecień S., Brzozowski T., Bubenik G.A., Pawlik W.W. (2007). Localization and biological activities of melatonin in intact and diseased Gastrointestinal Tract (GIT). J. Physiol. Pharmacol..

[81] Thaiss C.A., Levy M., Korem T., Dohnalová L., Shapiro H., Jaitin D.A., David E., Winter D.R., Gury-BenAri M., Tatirovsky E. (2016). Microbiota Diurnal Rhythmicity Programs Host Transcriptome Oscillations. Cell.

[82] Hsu P.Y., Harmer S.L. (2014). Wheels within wheels: The plant circadian system. Trends Plant Sci..

[83] Hennessey T.L., Field C.B. (1991). Circadian Rhythms in Photosynthesis: Oscillations in Carbon Assimilation and Stomatal Conductance under Constant Conditions. Plant Physiol..

[84] Dodd A.N., Salathia N., Hall A., Kévei E., Tóth R., Nagy F., Hibberd J.M., Millar A.J., Webb A.A.R. (2007). Plant Circadian Clocks Increase Photosynthesis, Growth, Survival. Science.

[85] Graf A., Schlereth A., Stitt M., Smith A.M. (2010). Circadian control of carbohydrate availability for growth in *Arabidopsis* plants at night. Proc. Natl. Acad. Sci. USA.

[86] Green R.M., Tingay S., Wang Z.-Y., Tobin E.M. (2002). Circadian rhythms confer a higher level of fitness to *Arabidopsis* plants. Plant Physiol..

[87] Hubbard C.J., Brock M.T., Van Diepen L.T., Maignien L., Ewers B.E., Weinig C. (2018). The plant circadian clock influences rhizosphere community structure and function. ISME J..

[88] Zhang C., Xie Q., Anderson R.G., Ng G., Seitz N.C., Peterson T., McClung C.R., McDowell J.M., Kong D., Kwak J.M. (2013). Crosstalk between the Circadian Clock and Innate Immunity in *Arabidopsis*. PLoS Pathog..

[89] Roenneberg T., Merrow M. (2016). The circadian clock and human health. Curr. Biol..

[90] Margulis L., Fester R. (1991). Symbiosis as a Source of Evolutionary Innovation.

[91] Simon J.C., Marchesi J.R., Mougel C., Selosse M.A. (2019). Host-microbiota interactions: From holobiont theory to analysis. Microbiome.

[92] Hörnlein C., Confurius-Guns V., Stal L.J., Bolhuis H. (2018). Daily rhythmicity in coastal microbial mats. NPJ Biofilms Microbiomes.

[93] D’Souza G., Shitut S., Preussger D., Yousif G., Waschina S., Kost C. (2018). Ecology and evolution of metabolic cross-feeding interactions in bacteria. Nat. Prod. Rep..

[94] Spengler M.L., Artemicheva N.M., Kuropatwinski K.K., Gasparian A.V., Fedtsova N., Gitlin I.I., Antoch M.P., Gudkov A.V., Deluca K.A., Comas M. (2012). Core circadian protein CLOCK is a positive regulator of NF-κB-mediated transcription. Proc. Natl. Acad. Sci. USA.

[95] Zhuang X., Magri A., Hill M., Lai A.G., Kumar A., Rambhatla S.B., Donald C.L., Lopez-Clavijo A.F., Rudge S., Pinnick K. (2019). The circadian clock components BMAL1 and REV-ERBα regulate flavivirus replication. Nat. Commun..

[96] Edgar R.S., Stangherlin A., Nagy A.D., Nicoll M.P., Efstathiou S., O’Neill J.S., Reddy A.B. (2016). Cell autonomous regulation of herpes and influenza virus infection by the circadian clock. Proc. Natl. Acad. Sci. USA.

[97] Dijk D.J., Duffy J.F., Silva E.J., Shanahan T.L., Boivin D.B., Czeisler C.A. (2012). Amplitude reduction and phase shifts of Melatonin, cortisol and other circadian rhythms after a gradual advance of sleep and light exposure in humans. PLoS ONE.

[98] Merrow M.W. (2009). Process for the Production of a Compound or a Composition Employing a Culture of Microorganisms under Circadian Cultivation Conditions. Patent Application.

[99] Vejan P., Abdullah R., Khadiran T., Ismail S., Nasrulhaq Boyce A. (2016). Role of plant growth promoting rhizobacteria in agricultural sustainability—A review. Molecules.

[100] Rudrappa T., Czymmek K.J., Pare P.W., Bais H.P. (2008). Root-Secreted Malic Acid Recruits Beneficial Soil Bacteria. Plant Physiol..

[101] Allard-Massicotte R., Tessier L., Lécuyer F., Lakshmanan V., Lucier J.F., Garneau D., Caudwell L., Vlamakis H., Bais H.P., Beauregard P.B. (2016). *Bacillus subtilis* early colonization of Arabidopsis thaliana roots involves multiple chemotaxis receptors. MBio.

[102] Beauregard P.B., Chai Y., Vlamakis H., Losick R., Kolter R. (2013). *Bacillus subtilis* biofilm induction by plant polysaccharides. Proc. Natl. Acad. Sci. USA.

[103] Qiao J., Yu X., Liang X., Liu Y., Borriss R., Liu Y. (2017). Addition of plant-growth-promoting *Bacillus subtilis* PTS-394 on tomato rhizosphere has no durable impact on composition of root microbiome. BMC Microbiol..

[104] Swain M.R., Ray R.C. (2009). Biocontrol and other beneficial activities of *Bacillus subtilis* isolated from cowdung microflora. Microbiol. Res..

[105] Sharaf-Eldin M., Elkholy S., Fernández J.A., Junge H., Cheetham R., Guardiola J., Weathers P. (2008). *Bacillus subtilis* FZB24^®^ affects flower quantity and quality of saffron (*Crocus sativus*). Planta Med..

[106] Hong C., Holtz B.A., Morgan D.P., Michailides T.J. (1997). Significance of thinned fruit as a source of the secondary inoculum of Monilinia fructicola in California nectarine orchards. Plant Dis..

[107] Ongena M., Jacques P., Touré Y., Destain J., Jabrane A., Thonart P. (2005). Involvement of fengycin-type lipopeptides in the multifaceted biocontrol potential of *Bacillus subtilis*. Appl. Microbiol. Biotechnol..

[108] Bais H.P., Fall R., Vivanco J.M. (2004). Biocontrol of *Bacillus subtilis* against infection of *Arabidopsis* roots by *Pseudomonas syringae* is facilitated by biofilm formation and surfactin production. Plant Physiol..

[109] Hanano S., Domagalska M.A., Nagy F., Davis S.J. (2006). Multiple phytohormones influence distinct parameters of the plant circadian clock. Genes Cells.

[110] Haydon M.J., Mielczarek O., Robertson F.C., Hubbard K.E., Webb A.A.R. (2013). Photosynthetic entrainment of the *Arabidopsis thaliana* circadian clock. Nature.

[111] Frank A., Matiolli C.C., Viana A.J.C., Hearn T.J., Kusakina J., Belbin F.E., Wells Newman D., Yochikawa A., Cano-Ramirez D.L., Chembath A. (2018). Circadian Entrainment in *Arabidopsis* by the Sugar-Responsive Transcription Factor bZIP63. Curr. Biol..

[112] Thain S., Murtas G., Lynn J. (2002). The circadian clock that controls gene expression in *Arabidopsis* is tissue specific. Plant Physiol..

[113] James A.B., Monreal J.A., Nimmo G.A., Kelly C.L., Herzyk P., Jenkins G.I., Nimmo H.G. (2008). The circadian clock in *Arabidopsis* roots is a simplified slave version of the clock in shoots. Science.

